# Evolutionary design of molecules based on deep learning and a genetic algorithm

**DOI:** 10.1038/s41598-021-96812-8

**Published:** 2021-08-27

**Authors:** Youngchun Kwon, Seokho Kang, Youn-Suk Choi, Inkoo Kim

**Affiliations:** 1 Samsung Advanced Institute of Technology, Samsung Electronics Co. Ltd., 130 Samsung-ro, Yeongtong-gu, Suwon-si, Gyeonggi-do 16678 Republic of Korea; 2 Department of Industrial Engineering, Sungkyunkwan University, 2066 Seobu-ro, Jangan-gu, Suwon-si, Gyeonggi-do 16419 Republic of Korea; 3 Data and Information Technology Center, Samsung Electronics Co. Ltd., 1-2 Samsungjeonja-ro, Hwaseong-si, Gyeonggi-do 18448 Republic of Korea

**Keywords:** Cheminformatics, Materials chemistry, Organic chemistry, Drug discovery, Chemistry, Materials science, Mathematics and computing, Optics and photonics

## Abstract

Evolutionary design has gained significant attention as a useful tool to accelerate the design process by automatically modifying molecular structures to obtain molecules with the target properties. However, its methodology presents a practical challenge—devising a way in which to rapidly evolve molecules while maintaining their chemical validity. In this study, we address this limitation by developing an evolutionary design method. The method employs deep learning models to extract the inherent knowledge from a database of materials and is used to effectively guide the evolutionary design. In the proposed method, the Morgan fingerprint vectors of seed molecules are evolved using the techniques of mutation and crossover within the genetic algorithm. Then, a recurrent neural network is used to reconstruct the final fingerprints into actual molecular structures while maintaining their chemical validity. The use of deep neural network models to predict the properties of these molecules enabled more versatile and efficient molecular evaluations to be conducted by using the proposed method repeatedly. Four design tasks were performed to modify the light-absorbing wavelengths of organic molecules from the PubChem library.

## Introduction

The discovery of new functional molecules has led to many technological advances and is still one of the most crucial ways in which to overcome technical issues in various industries, such as those in the organic semiconductor, display, and battery industries. Although the trial-and-error approach has generally been considered as the most acceptable way to develop new materials, computer-aided techniques are increasingly being used to enhance the efficiency and hit rate of molecular design^1^. A typical example is high-throughput computational screening (HTCS), which involves the use of virtual chemical libraries for large-scale predictions of material properties using simulations or machine learning, allowing a rational sorting of potential candidates for subsequent chemical synthesis^2,3^. However, HTCS is a local optimization technique whose success relies on the quality of the chemical libraries, the development of which depends on researchers’ experience and intuition. Thus, HTCS has a low hit rate, and in most cases, several iterative enumerations are necessary to generate suitable target materials. In this regard, evolutionary algorithms, a type of exhaustive enumeration, can be a viable alternative for de novo design. These algorithms are generic population-based metaheuristic optimization techniques that use bio-inspired operators, such as reproduction, mutation, recombination, and selection^4,5^. As design tools for materials, they not only optimize the molecular structures but also provide hints for a promising chemical space by identifying genetic traits that favor the target properties while maintaining the unique genotypes of ancestors. Recent advances in machine-learning algorithms^6–19^ have led to the proposal of data-driven methodologies.

The development processes of various materials, including organic molecules, metals, ceramics, composites, and carbon molecules^20,21^, have widely adopted evolutionary design methodologies (EDM) in combination with property prediction techniques^22–29^ based on first-principles calculation and machine learning. Specifically regarding organic molecules, two major challenges of EDM are to (1) preserve the chemical validity of evolved molecules and (2) choose the best-fit individuals in each generation efficiently and accurately according to the fitness function. To address the first challenge, heuristic chemical knowledge is generally incorporated. Molecules expressed as graphs or ASCII strings evolve according to user-defined rules, such as adding, deleting, and replacing atoms, bonds, and substructures under chemical constraints. Notably, not only the fragment structures that serve as building blocks but also their attachment points are specified in advance based on previous experience. This method increases the likelihood of generating more valid and synthetically tractable molecules and sometimes accelerates overall stochastic searches by using in-depth domain knowledge. However, predefined chemical rules and fragment libraries can lead to bias, and therefore the entire optimization process is at risk of converging to local optima. Moreover, every time the application changes, new chemical rules would have to be specified. For some emerging areas, it is challenging to determine a well-established guide for structural changes. To address the second challenge, simple evaluation methods, such as structural similarity indices, quantitative structure–property relationship models, and semi-empirical quantum chemistries, are generally adopted as fitness functions to reduce the computational cost. However, more diverse and complex assessments are needed to evaluate the candidates precisely but promptly.

To overcome these demanding limitations, we devised an evolutionary molecular design method based on deep learning. Instead of graphs or ASCII strings, a bit-string fingerprint vector is used as a molecular descriptor to evolve molecules. Then, the evolved fingerprint vectors are converted into actual molecular structures using a recurrent neural network (RNN) model^30^, which acts as a decoder. This approach enables us to prevent explicit chemical knowledge from intervening during a molecular evolution while ensuring the molecules are chemically valid. Moreover, deep neural network (DNN) models^31^, aided by quantum chemical calculations, are used to evaluate the evolved molecules with more complex criteria.

The effectiveness of the entirely data-driven evolutionary approach was validated by conducting various molecular design tasks on data in the PubChem library to change the wavelengths at which organic molecules absorb the maximum amount of light^32^. The deep learning models learn implicit knowledge from this rich library of materials and successfully guide the automatic evolution of seed molecules without heuristic intervention.

## Computational methods

### Workflow of the evolutionary design

**Figure 1:**
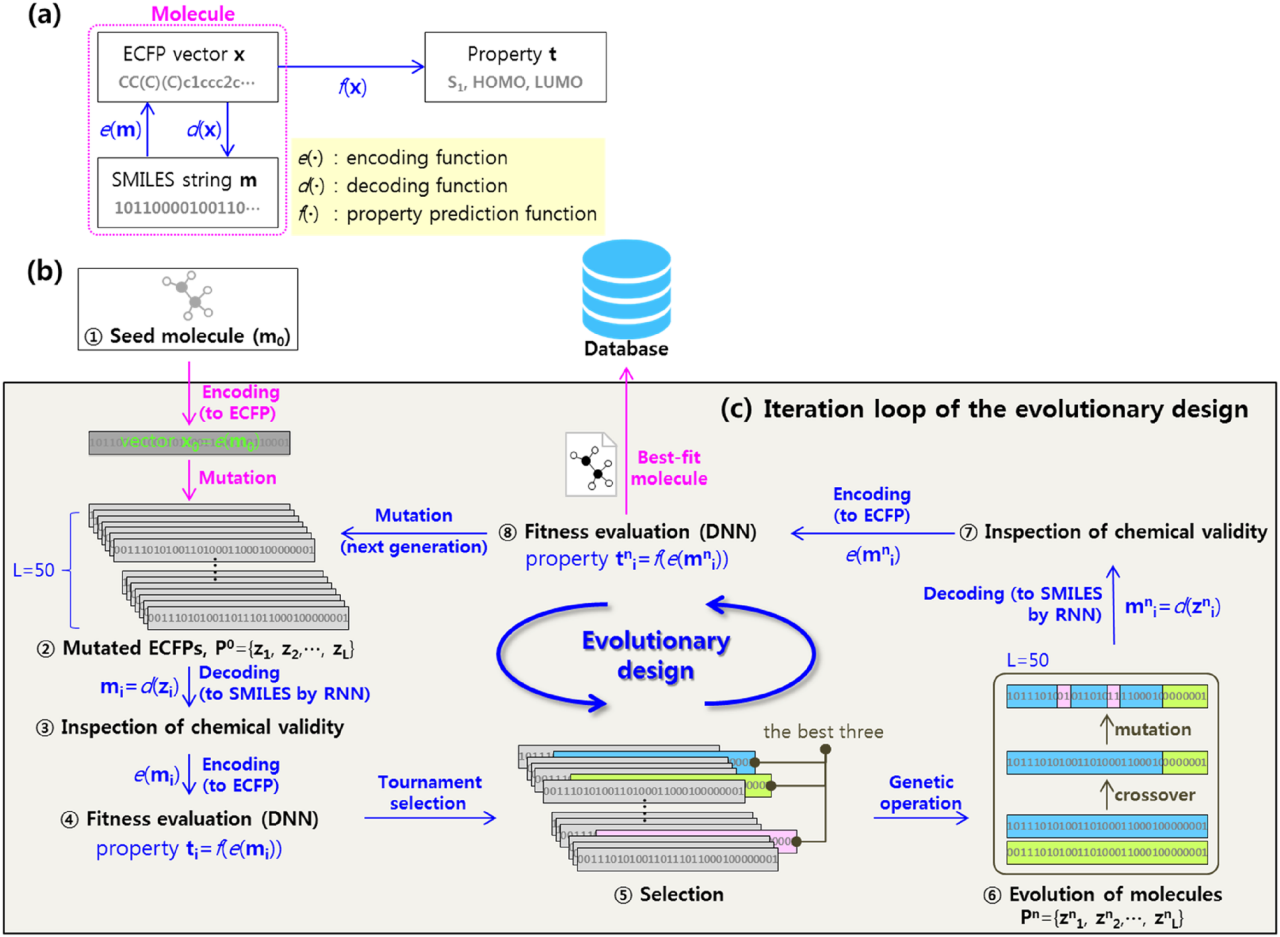
Schematics of the deep learning-based evolutionary molecular design. (**a**) Molecular representations and their relationships with the encoding, decoding, and property prediction functions. (**b**) Detailed workflow of the evolutionary design. (**c**) Iteration loop of the evolutionary design.

The evolutionary design framework where a genetic algorithm (GA) finds the design route towards the target under the guidance of deep learning models is illustrated in Fig. 1. This approach automatically optimizes the structure of seed molecules via the collaborative work of an encoding function *e*(**∙**), decoding function *d*(**∙**), and property prediction function *f*(**∙**). As illustrated in Fig. 1a, the encoding function *e*(**∙**) transforms the molecular structure **m**, which exists in a canonical simplified molecular input line entry system (SMILES)^33^ string format, into the corresponding extended-connectivity fingerprint (ECFP)^34^ vector **x**. Then, the decoding function *d*(**∙**) converts the bit-string ECFP vector **x** into the SMILES string **m** to enable it to be recognized as a real chemical structure. The property prediction function *f*(**∙**) predicts the molecular property **t** using the ECFP vector as an input. The decoding and property prediction functions are derived by the RNN and DNN, respectively, and consequently, lead the overall workflow shown in Fig. 1b.

First, the molecular structure of a seed molecule **m**_**0**_ in SMILES format is transformed into the corresponding ECFP vector **x**_**0**_ using the encoding function *e*(**∙**). Then, the evolution procedure begins with the generation of a population of vectors **P**^**0**^ = {**z**_**1**_, **z**_**2**_,∙…∙, **z**_**L**_} through the mutation of **x**_**0**_. Following the conversion of each vector **z**_**i**_ into a SMILES string **m**_**i**_ by *d*(**z**_**i**_), the validity of the decoded SMILES strings is inspected in terms of grammatical correctness with the RDKit library, e.g., additional open/close parentheses, unclosed rings, and Kekulization feasibility. This serves to evaluate the fitness of the molecules by predicting the molecular properties with **t**_**i **_= *f*(*e*(**m**_**i**_)). Subsequently, the top three ECFP vectors based on fitness are selected as parents for further evolution into the new population of vectors **P**^**n**^ = {**z**^**n**^_**1**_, **z**^**n**^_**2**_,∙…∙, **z**^**n**^_**L**_} via crossover and mutation. Notably, the assessment of the chemical validity and fitness evaluation are performed once again, and the best-fit molecule in the *n*^th^ generation is chosen. Generated molecules that overlap with molecules that already exist in the database are removed from the candidate list. Successive iterations of this procedure gradually refine the properties and automatically optimize the molecular structures to meet the target.

During the evolution, additional constraints, such as the presence or absence of specific substructures, can be imposed on the molecular structure depending on the design purpose. However, in this study, three simple structural constraints were specified in the form of a blacklist. Newly generated molecular structures that included these blacklisted substructures were then excluded from the list of candidate materials. The first constraint specifies that the size of fused rings in molecules generated with such rings be limited to between four and seven. Second, the maximum number of fused rings in the molecules is restricted to six, and finally the maximum length of an alkyl chain is limited to six carbon atoms. Third, to maintain the form of the seed molecule, we remove the following newly generated molecules: those for which the maximum number of rings exceeds those of the seed molecule by two and those for which the minimum number of rings is two fewer than those in the seed molecule. These structural restrictions made it possible to obtain novel molecules with appropriate properties via natural evolution without significantly deviating from the shape of the initial seed.

### Encoding, decoding, and property prediction functions

ECFP is a circular topological fingerprint that has been successfully applied to represent organic molecules in vector forms. The encoding function *e*(**∙**) uses a hash function to map the structural features of a molecule into a fixed-length ECFP vector representation. In this study, we employ an ECFP with a neighborhood size of 6 and a length of 5000. Therefore, the encoding function encodes each atom and its circular neighborhoods with a diameter of six chemical bonds for a molecule **m** and transforms the SMILES into a 5000-dimensional vector **x**. Regarding the decoding function *d*(**∙**), an RNN composed of three hidden layers with 500 long short-term memory units^35^ is modeled to obtain the SMILES string from the ECFP vector. SMILES represents a molecular structure as a compact variable-length sequence of characters using simple vocabulary and grammar rules. As proven in recent studies, the RNN can generate SMILES strings because it effectively captures the long-term dependence of sequences. This occurs via the recurrent connections of units across the sequence steps. We form an RNN as a language model that generates a single-step moving window sequence of three-character substrings for each SMILES string. Here, the next substring in the sequence is predicted by conditioning the current substring and the given ECFP vector. This conditional generation of three-character substrings usually reduces the ratio of invalid SMILES by imposing additional restrictions on the subsequent character. The grammatically incorrect SMILES strings are deleted in the inspection step.

To obtain the property prediction function *f*(**∙**), a five-layer DNN was built with 250 hidden units in each layer to identify the nonlinear relationship between molecular structures and their properties^36^.

In the RNN, the output layer is a softmax activation function to indicate the probability distribution of substrings. As for a DNN, all hidden layers use a logistic sigmoid activation function, and the output layer employs a linear function. The inputs of the RNN and DNN are 5000-dimensional ECFP vectors. All neural networks are trained by backpropagation using the Adam optimizer^37^ with a mini-batch size of 100 and 500 training epochs. Each input and hidden layer in the neural networks is followed by a dropout layer with a dropout rate of 0.5 to prevent overfitting. Suppose that a dataset of *k* molecules and their annotated properties ***D* = **{(**m**_**i**_, **t**_**i**_)}_i=*1–k*_ is given. Subsequently, the RNN is trained on ***D***^RNN^ = {(*e*(**m**_**i**_), **m**_**i**_)}_i=*1–k*_ by minimizing the cross entropy between the softmax output and **m**_**i**_, aiming at fulfilling the functional relationship *d*(*e*(**m**_**i**_)) = **m**_**i**_. In addition, the DNN is trained on ***D***^DNN^ = {(*e*(**m**_**i**_), **t**_**i**_)}_i=*1–k*_ by minimizing the mean squared error between *f*(*e*(**m**_**i**_)) and **t**_**i**_. All neural networks were implemented using the Keras library based on the GPU-accelerated Theano library.

### Genetic algorithm

The GA procedure was implemented using the Distributed Evolutionary Algorithms (DEAP) library in Python. The size of the population, crossover rate, and mutation rate are set to 50, 0.7, and 0.3, respectively. Following an initial mutation in each generation (**P**^**0**^ in Fig. 1b), a tournament selection with a size of 3 is conducted to select parents for further evolution with crossover and mutation. For the former, we used a uniform crossover with a mixing ratio of 0.2 between two parent individuals. For the latter, we used Gaussian mutation that adds random values drawn from *N*(0, 0.2^2^) to elements chosen with a ratio of 0.01 in an individual ECFP vector. The overall evolution was terminated when (1) the number of generations reached 500 and (2) the fitness was not enhanced during 30 consecutive generations. The default values in the DEAP library were used for the additional settings.

### Quantum chemistry

All quantum chemical calculations were performed with the Gaussian 09 program suite^38^. The molecular geometries were optimized by density functional theory (DFT) using the hybrid B3LYP functional and all-electron 6-31G basis sets. A single-point time-dependent DFT calculation was performed with this geometry to calculate the vertical excitation energies to the lowest singlet state (S_1_). Symmetry constraints were not imposed in any of the calculations.

## Results and discussion

### Performance of deep neural networks

The effectiveness of the deep learning-based evolutionary design was verified by applying it to real-world problems. The aim was to change the maximum light-absorbing wavelengths in terms of the S_1_ energy. Accordingly, the RNN and DNN models were trained with a chemical library that comprises 10,000 to 100,000 molecules (with molecular weights between 200 and 600 g/mol) randomly sampled from the PubChem database^32^. Each molecule was labeled with the excitation energy (S_1_), molecular orbital energies (highest occupied molecular orbital (HOMO), and lowest unoccupied molecular orbital (LUMO)) by the DFT calculation. As the amount of training data increases, the performance of the RNN and DNN models improves accordingly, as summarized in Table 1. In the case of 100,000 training data points, the validity of RNN decoding was 88.8%, the reconstructability was 67.4% and the correlation coefficients between the DNN predictions and simulated values of S_1_, HOMO, and LUMO were 0.977, 0.948, and 0.96, respectively, in a tenfold cross-validation (also refer to Fig. S1 in the Supplementary material). The dataset was partitioned into training (90%) and test sets (10%). For each data split, we trained the prediction model using the training set and evaluated its prediction performance on the test set. The validity of RNN decoding, which refers to the proportion of chemically valid molecules, was assessed during the RDKit inspection step. In the case of reconstructability of RNN decoding, input descriptor was evaluated by trying to retrieve the molecules that was represented by them. By identifying the sampled canocnical SMILES string in 10,000 generated strings given seed molecules from the test dataset, almost 62.4% of the consisted of strings with the same canonical form as the molecule behind the seeding ECFP.

**Table 1:** Performance of DNN based on the number of training data. *R* is the correlation coefficient between DNN predictions and simulated values, and MAE (eV) is the mean absolute error in the tenfold cross-validation.

Number of training data	Validity (%)	Reconstructability (%)	Prediction accuracy of DNN (R, MAE)		
			S_1_	HOMO	LUMO
100,000	88.8	62.4	0.977, 0.185	0.948, 0.168	0.960, 0.195
50,000	86.7	60.1	0.973, 0.198	0.945, 0.172	0.955, 0.209
30,000	85.3	59.8	0.930, 0.228	0.934, 0.191	0.945, 0.224
10,000	83.2	55.7	0.913, 0.278	0.885, 0.244	0.917, 0.287

### Evolutionary design for S_1_ change without any constraints

**Figure 2:**
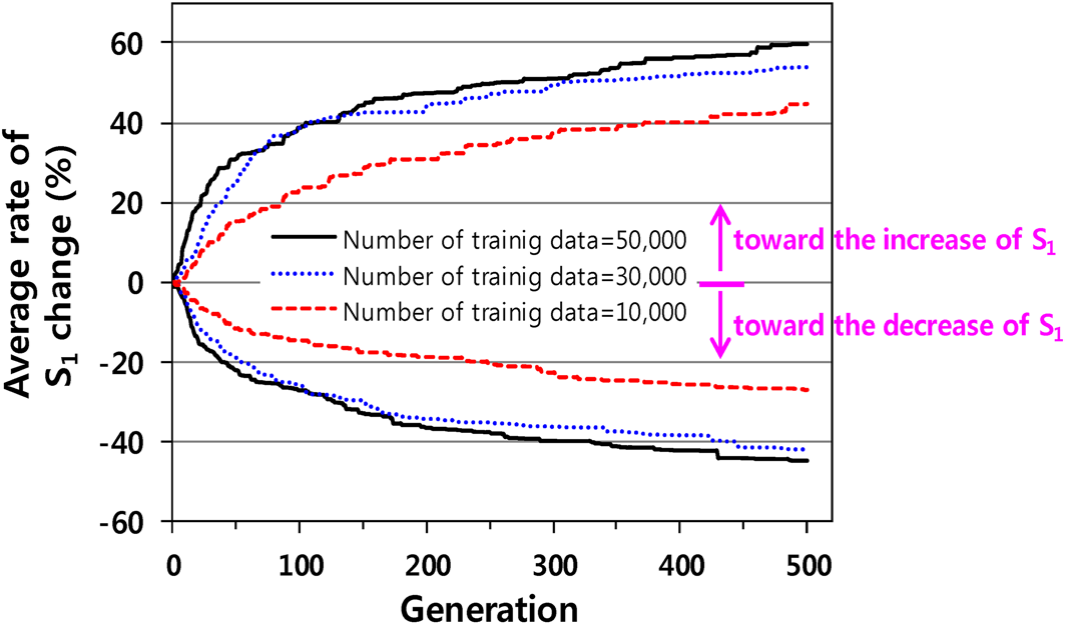
Average rates of change of S_1_ for the 50 seed molecules during the evolutionary design involving increasing and decreasing S_1_.

Fifty seed molecules were randomly selected from the chemical library and evolved in both.

directions by increasing and decreasing S_1_. The S_1_ values of the seed molecules are between 3.8 eV and 4.2 eV. Figure 2 shows the average rate of change of S_1_ for the 50 seed molecules when the number of training data samples increases from 10,000 to 50,000. The average fitness improves with the number of generations when S_1_ is increased or decreased, indicating that the proposed workflow has successfully evolved the seed molecules toward those with the required target properties. In the early stage, S_1_ changes fast, where after the change is relatively slow. Moreover, a larger amount of training data results in a higher rate of S_1_ change.

In the increasing direction, the average S_1_ increases by approximately 60% for 50,000 training data; however, it increases by merely 45% for 10,000 training data. This seems to suggest that these results reflect the difference in the performance of the RNN models. The RNN model trained with a larger dataset learns more decoding rules and would decode fingerprints into more diverse molecular structures to meet the goals. Overall, the evolution trend tends to become almost saturated when approximately 50,000 training data samples are used. Thus, in this design circumstance, 50,000 data samples are sufficient to train deep learning models.

In the decreasing direction, the maximum rates of change in S_1_ are slightly lower than those in the increasing direction, which may be caused by the S_1_ distribution of the training data. As shown in Fig. 2, in the case of 50,000 samples of training data, the S_1_ distribution is skewed and is higher than the median S_1_ value of the seed molecules, i.e., 4.0 eV. The average S_1_ values are 4.4, 4.3, and 4.4 eV for 10,000, 30,000, and 50,000 samples of training data, respectively. Owing to the characteristics of the training data, S_1_ is more likely to change its value in the increasing direction. Although not included in Fig. 2, the S_1_, HOMO, and LUMO distributions for 10,000 and 30,000 samples of training data are similar to those of the 50,000 samples of training data.

### Evolutionary design for S_1_ change using the HOMO and LUMO as constraints

In an additional design task, we evolve molecules by using the HOMO and LUMO energies as constraints. The seed molecules are the same as before, and the amount of training data is fixed at 50,000 samples. We apply the constraints of − 7.0 eV < HOMO <  − 5.0 eV and LUMO < 0.0 eV for the generated molecules.

**Figure 3:**
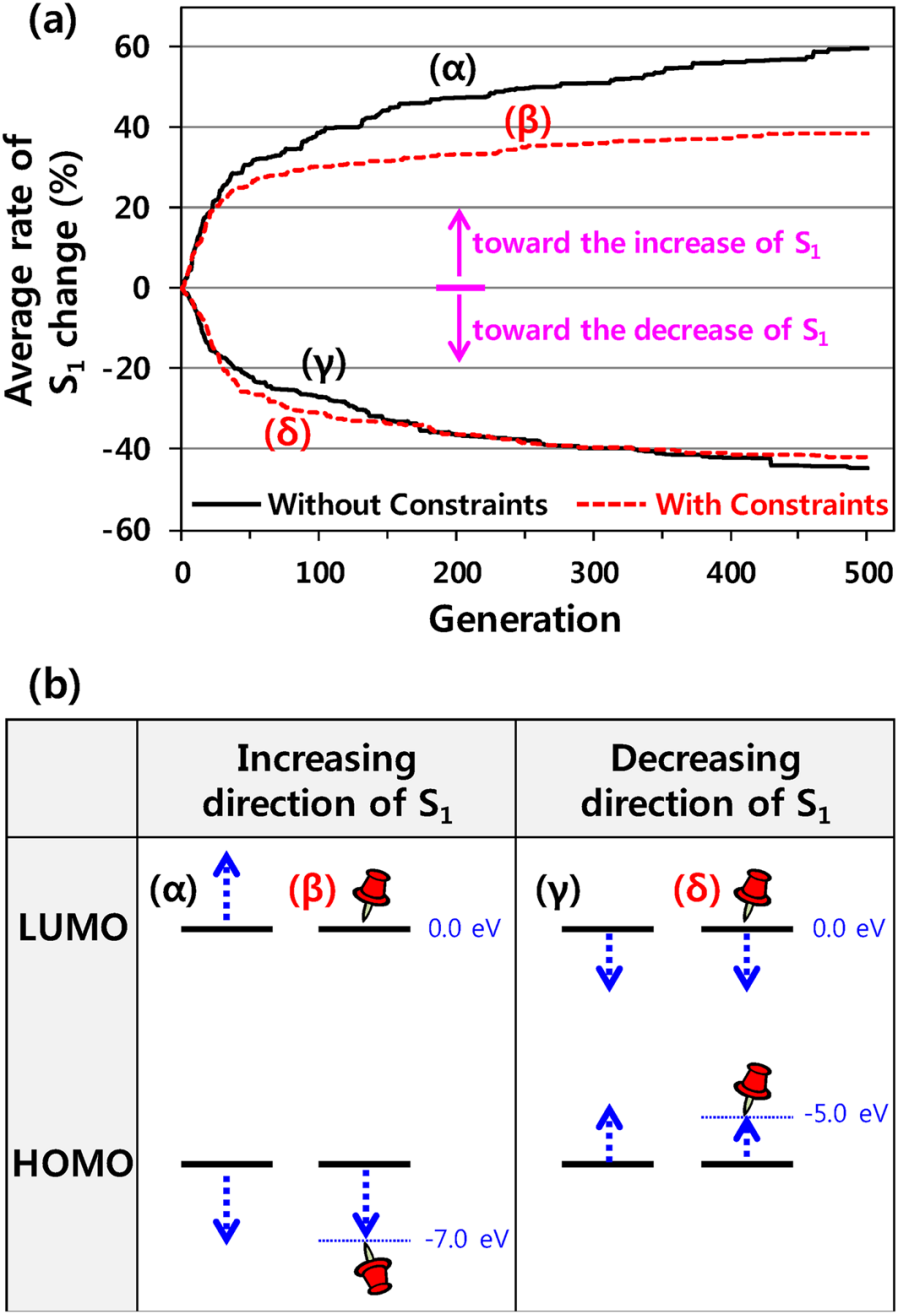
Effect of HOMO and LUMO constraints on the evolutionary design. (**a**) Average rates of change of S_**1**_ for the 50 seed molecules. (**b**) Schematics of the change in the molecular orbital energy when S_**1**_ is increased and decreased.

In the increasing direction of S_1_, the molecules that evolve without any constraints exhibit higher rates of S_1_ change than those that evolve within the constraints, as shown in Fig. 3a. Generally, S_1_ is proportional to the energy gap between HOMO and LUMO. Under the constraints, the maximum LUMO is fixed at 0.0 eV, as depicted in Fig. 3b. Therefore, increasing S_1_ requires the HOMO energy to be lowered. However, the minimum HOMO energy is also limited to − 7.0 eV. These constraints are therefore responsible for suppressing the increase in S_1_.

Interestingly, the use of constraints does not significantly affect the rate at which S_1_ is decreased. To decrease S_1_, the energy difference between HOMO and LUMO must be reduced. Under the constraints, LUMO is still assigned a maximum value of 0.0 eV, as delineated in Fig. 3b; however, LUMO can freely move in a downward direction. Moreover, although the maximum HOMO is limited to − 5.0 eV, the distributions of the training data in Fig. S2 and indicate that the constraints allow sufficient room in which to decrease the energy gap between HOMO and LUMO. The constraints in the form of the HOMO and LUMO energies thus have the opposite effect on the increasing and decreasing S_1_ energy.

**Figure 4:**
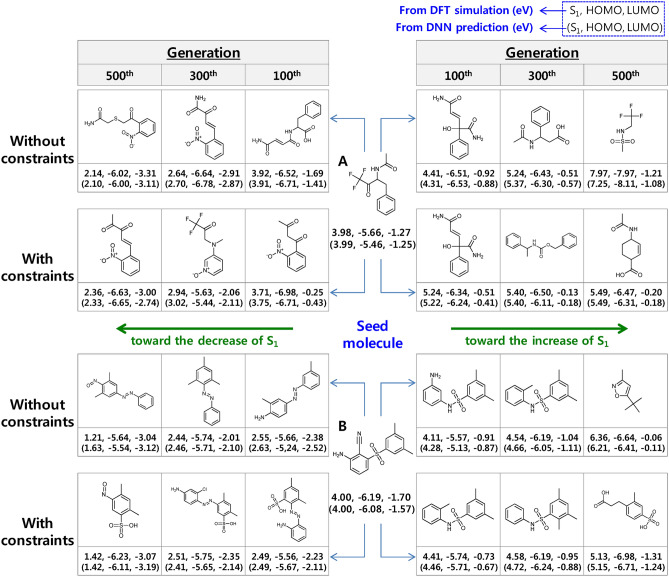
Examples of evolved molecules for two seed molecules (**A**, **B**) in the absence and presence of constraints. The DFT-simulated and DNN-predicted (in parentheses) energy values are annotated together.

Examples of the molecules that evolved from two seed molecules, in the absence and presence of the constraints, are summarized in Fig. 4. Local exceptions occurred in a few of the generations. Nevertheless, the overall evolution proceeds as anticipated. The molecules evolve through structural modifications, such as the addition, deletion, and substitution of atoms and substructures. As the number of generations increases, the structural changes accumulate, and a wider variety of moieties are introduced towards attaining the target property.

As a result, the deep learning-based functions, *d*(**∙**) and *f*(**∙**), enable successful molecular evolution by acquiring the knowledge latent in the molecular data. In most cases, multiple evolutions of the same seed molecule occur along different design paths owing to the randomness of GA. Therefore, more diverse offspring can be obtained using an iterative approach.

### Evolutionary molecular design outside the scope of the training data

**Figure 5:**
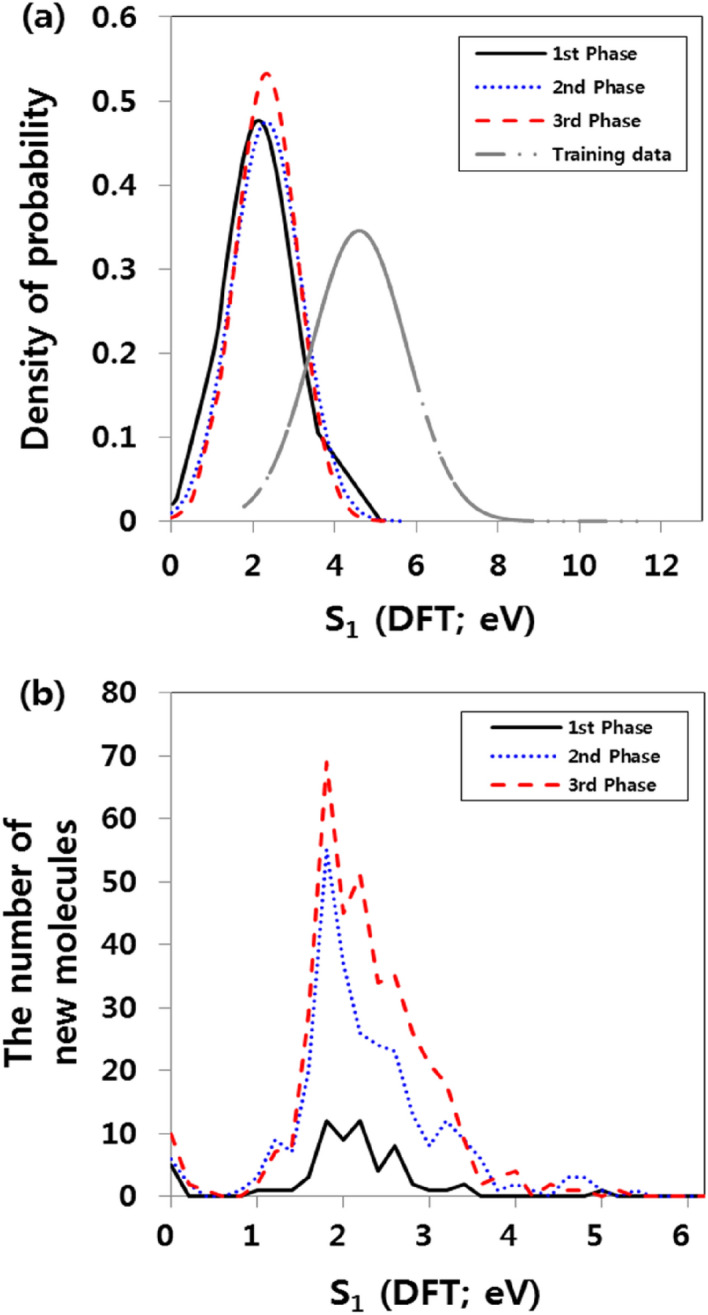
Percentage of molecules generated with evolutionary design vs. the density of the training dataset (**a**) and the number of new molecules generated in repeated phases (**b**).

The data-driven approach is heavily influenced by the training data. The challenge would therefore be to obtain a group of molecules outside the scope of the specified target property. As a final experiment, to generate a molecular structure with properties in the extrapolation area, we added a process that repeatedly calculates newly generated molecules and re-trains the RNN and DNN models. To create a group of molecules with S_1_ values smaller than 1.77 eV using data with an S_1_ distribution above 1.77 eV, we selected the 30 molecules with the smallest S_1_ values in the training data as seed molecules. Based on the sampled 30 molecular seeds, the process of generating new molecules was repeated 300 times to derive new molecules with S_1_ lower than 1.77 eV. We calculated the new molecules by DFT and then re-trained the RNN and DNN models, similar to the initial training process. We defined this iterative process as a “phase” and repeated it three times. When proceeding with the next phase, we selected the 30 molecules with the lowest S_1_ values as the seed, including the new molecules created in the previous phase. This yielded an average S_1_ value for the training data of 4.91 eV, and the variance is 2.11. However, the aforementioned process produces new molecules for which the S_1_ distribution is relatively lower than that of the training data, as shown in Fig. 5a. The average S_1_ of the molecules produced in the first phase is 2.20, and the variance is 1.40. The averages in the second and third phases are 2.22 and 2.31, with variances of 1.38 and 1.36, respectively. Although the average value rises slightly as the number of phases increases, the variance gradually decreases. This intensifies the creation of molecular structures with the desired physical properties.

**Figure 6:**
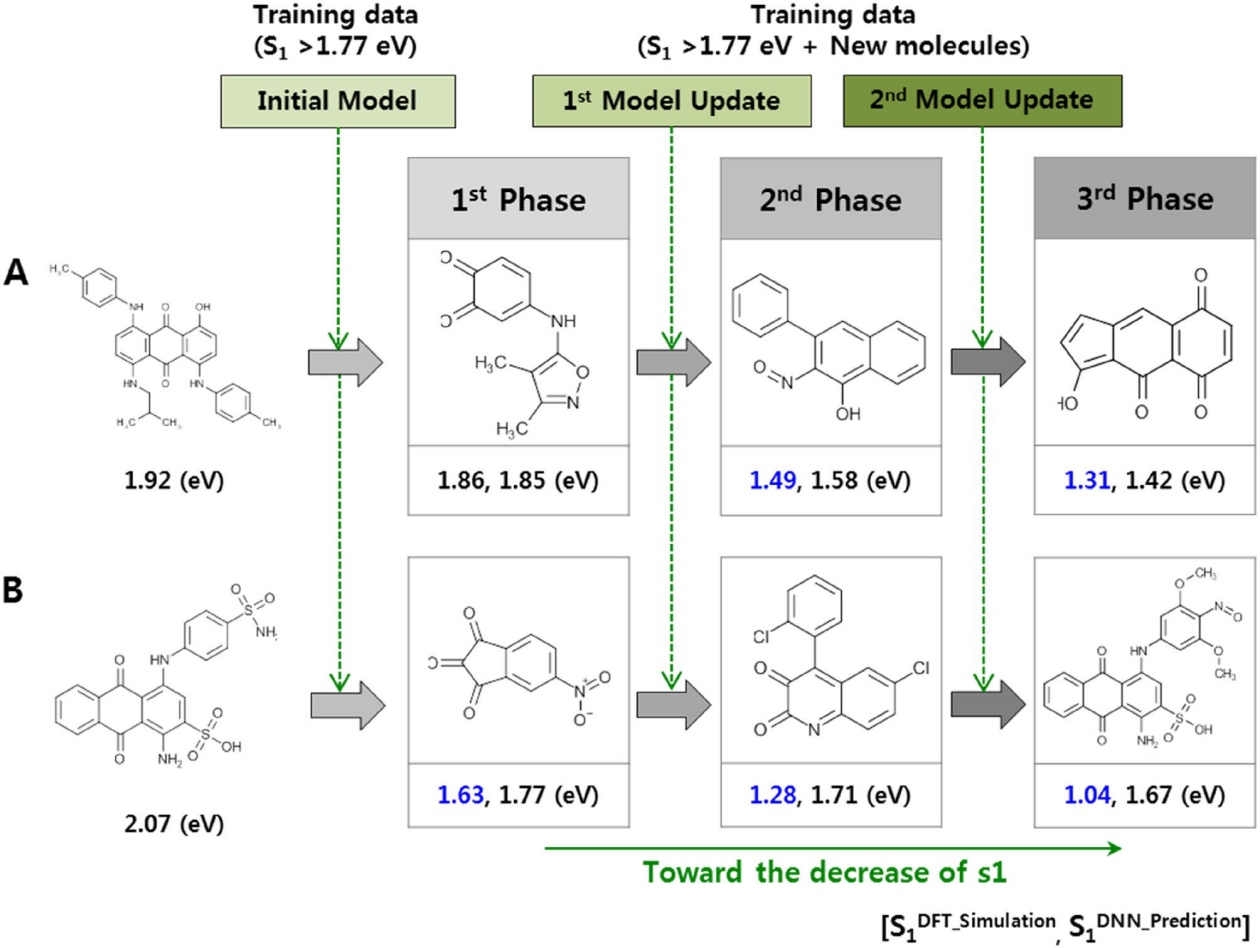
Two examples of the process of keeping the shape of the initial seed molecules while exploring the training data of the properties of S_1_.

As shown in Fig. 5b, as the number of phases increases, the number of new molecules with S_1_ values that are relatively lower than those in the previous phase increases. After the first phase, the number of newly generated molecules with S_1_ values lower than 1.77 eV is 12. In the second and third phases, this number becomes 37 and 58, respectively. This process makes it possible to secure a group of potential new materials with properties that are not in the training data without significant changes in the shape of the molecular structure (Fig. 6). The predicted accuracy is low because of the relatively small number of molecules with very low S_1_ values in the overall data distribution. However, as the process continues to accumulate sufficient data, the predicted accuracy is likely to increase, and this would enable candidate materials with new values to be obtained.

### Goal-Directed Benchmarks

We compared the proposed method with previous generative models by testing it on goal-directed tasks defined in the GuacaMol^8,12^ scoring suite. The comparison involved using the proposed method to generate novel molecules with the desired properties. The two main objectives of validation of the GuacaMol test are rediscovery and property satisfaction benchmarks. Specifically, the rediscovery task is defined as the maximization of the similarity between the ECFP fingerprints of the structures of the generated molecules and that of the target. We employed celecoxib, troglitazone, and thiothixene as benchmarks for the rediscovery tasks, such as baselines. To apply the EDM model to the GuacaMol task, we newly trained our RNN model using the ChEMBL25 training dataset^12^. Unlike baselines such as cRNN^12^, the EDM has to redefine the range of characters with which to train the RNN model because ours trains three consecutive characters of the SMILES string as one unit character. The baselines used prior knowledge in the form of the 100–300 known highest-scoring molecules from the ChEMBL dataset as initial points for the rediscovery task. They also adapted the target of interest via the scoring function. For fair comparison, the EDM model chooses the 256 highest-scoring molecules in the test dataset. The model then generates approximately 500 SMILES strings using the same conditional seed for each seed molecules, in which case the rediscovery is awarded a score of 1.0 for all three conditions.

Regarding the property satisfaction benchmarks, the EDM model was trained on the two logP targets, as well as on the topological polar surface area (TPSA), quantitative estimate of drug-likeness (QED), and the central nervous system multi-parameter optimization (CNS MPO) tasks. In addition, we also selected the 100 top-scoring molecules from the ChEMBL25 test dataset as conditional seed to compare with the baselines. The EDM model generated a single batch of 500 SMILES strings. The performance of the model was similar to that of the cRNN in that they generated SMILES strings by extracting the ECFP that satisfied the initially constrained properties. Overall, the validation results confirm that the EDM method delivers performance comparable to that of the cRNN and other algorithms by achieving the maximum score for all eight of the given tasks. These results are summarized in Table 2.

**Table 2:** Comparison of goal-directed benchmarks.

Benchmark	Best of dataset		SMILES LSTM		SMILES GA		Graph GA		Graph MCTS		cRNN		EDM (ours)
Celecoxib red	0.505		**1.000**		0.607		**1.000**		0.378		**1.000**		**1.000**
Troglitazone red	0.419		**1.000**		0.558		**1.000**		0.312		**1.000**		**1.000**
Thiothixene red	0.456		**1.000**		0.495		**1.000**		0.308		**1.000**		**1.000**
LogP(− 1.0)	**1.000**		**1.000**		**1.000**		**1.000**		0.980		**1.000**		**1.000**
LogP(8.0)	**1.000**		**1.000**		**1.000**		**1.000**		0.979		**1.000**		**1.000**
TPSA(150.0)	**1.000**		**1.000**		**1.000**		**1.000**		**1.000**		**1.000**		**1.000**
CNS MPO	**1.000**		**1.000**		**1.000**		**1.000**		**1.000**		**1.000**		**1.000**
QED	**0.948**		**0.948**		**0.948**		**0.948**		0.944		**0.948**		**0.948**

## Conclusions

An entirely data-driven evolutionary molecular design methodology based on deep learning models was developed in this study. In the proposed method, a GA along with RNN and DNN models were used to evolve the fingerprint vectors of seed molecules. The RNN decoder reconstructed chemically valid molecular structures in the SMILES format from the evolved fingerprint vectors without resorting to predefined chemical rules. In addition, the DNN efficiently evaluated the suitability of the evolved molecules even within a more complex range of properties.

The closed-loop evolutionary workflow guided by deep learning automatically and effectively derived target molecules and discovered rational design paths by elucidating the relationship between the structural features and their effect on the molecular properties. Furthermore, owing to the inherent nature of data-driven methodologies, the molecular design performance can be influenced by the characteristics of the training data. Therefore, the training data should be prepared carefully according to the design purpose and situation. Unlike the test cases used illustratively in this study, the data to train the RNN and DNN need not be the same and could perhaps be configured differently depending on the design target. Moreover, traditional generative models based on data-driven approaches have limited ability to design new molecules with properties that are not included in the training datasets. In contrast, the proposed method can be designed to produce a new group of candidates by repeating the generation and calculation in that direction even if the molecules with the desired range of chemical characteristics are not included in the training data.

Lately, various computer-aided techniques for designing materials, such as inverse designs, exhaustive enumerations, and molecular structure optimization models, have been proposed. Because each method has its advantages and disadvantages, the methods may act synergistically when used together rather than alone. In this respect, our evolutionary design method is also expected to be a promising tool with which to explore the enormous chemical space and facilitate the discovery of novel materials.

## Supplementary Information

Supplementary Information.
