# Supplementary material for: Evolutionary design of molecules based on deep learning and a genetic algorithm
Source: Sci Rep. 2021 Aug 27;11:17304. doi: 10.1038/s41598-021-96812-8 (PMC8397714; doi:10.1038/s41598-021-96812-8)
Supplement: Supplementary file 1 — Supplementary Information. [file 41598_2021_96812_MOESM1_ESM.docx]

**Supplementary Materials for**

**Evolutionary design of molecules based on deep learning and genetic algorithm**

Young Chun Kwon^§a^, Seokho Kang^§b^, Youn-Suk Choi*^a^, and Inkoo Kim^c^

*^a^ Samsung Advanced Institute of Technology, Samsung Electronics Co. Ltd., 130 Samsung-ro, Yeongtong-gu, Suwon-si, Gyeonggi-do 16678, Republic of Korea*

*^b^ Department of Systems Management Engineering, Sungkyunkwan University, 2066 Seobu-ro, Jangan-gu, Suwon-si, Gyeonggi-do 16419, Republic of Korea*

*^c^ Data and Information Technology Center, Samsung Electronics Co. Ltd, 1-2 Samsungjeonja-ro, Hwaseong-si, Gyeonggi-do 18448, Republic of Korea*

^§^ These two authors contributed equally to this work.

* Corresponding author. E-mail: ysuk.choi@samsung.com; Tel.: +82-31-8061-4387; Fax: +82-31-8061-1576.

**Fig. S1** Prediction accuracy of DNN models for (a) S1, (b) HOMO, and (c) LUMO using 10-fold cross-validation. *R* is the correlation coefficient between the DNN prediction and DFT simulation.

**Fig. S2** Energy distributions of (a) S_1_, (b) HOMO, and (c) LUMO in 50,000 training data.
